# Leveraging the potential of synthetic text for AI in mental healthcare

**DOI:** 10.3389/fdgth.2022.1010202

**Published:** 2022-10-24

**Authors:** Julia Ive

**Affiliations:** School of Electronic Engineering and Computer Science, Queen Mary University of London, London, UK

**Keywords:** synthetic text, natural language generation, mental health text, privacy protection, AI for mental health

## Abstract

In today’s world it seems fair to say that extensive digital data sharing is the price we pay for the technological advances we have seen achieved as a result of AI systems analysing large quantities of data in a relatively short time. Where such AI is used in the realm of mental health, this data sharing poses additional challenges not just due to the sensitive nature of the data itself but also the potential vulnerability of the data donors themselves should there be a cybersecurity data breach. To address the problem, the AI community proposes to use synthetic text preserving only the salient properties of the original. Such text has potential to fill gaps in the textual data availability (e.g., rare conditions or under-represented groups) while reducing exposure. Our perspective piece is aimed to demystify the process of generating synthetic text, explain its algorithmic and ethical challenges, especially for the mental health domain, as well as most promising ways of overcoming them. We aim to promote better understanding and as a result acceptability of synthetic text outside the research community.

## 1. Introduction

Over the last few years the use of AI has proved to be a boon for both mental health professionals and research into the field. For example, AI is actively used to analyse data from social-media platforms such as Twitter and Reddit to monitor for particular conditions (e.g., depression or suicidal ideation), analysing the language used in its therapy sessions to assist human specialists facing increased workload, improving personalised treatment plans and in training professionals (1–3). Indeed, AI shows strong potential to improve both mental health research and practice through learning from relevant patterns in specimen textual datasets. However, such datasets are not always easily available and are very often costly to obtain (aka, data *sparsity* problem, e.g., it may take years to obtain longitudinal mental health data to adequately monitor disease progression). More importantly, the usage of such data in AI raises serious *privacy* considerations (4). Those are not only due to the sharing of sensitive mental health text but also due to the fact that AI models internally memorise their training data which could pose cybersecurity exposures once they are deployed (5,6).

An approach gaining ground in the AI community to address these issues of data sparsity and privacy is by producing and using synthetic data, including textual data, to reduce the reliance on human derived data. We need to emphasise here that privacy is a major limiting factor affecting data availability. Resolving, or at least significantly assuaging privacy concerns will ease this data sparsity constraint. Relevant research investigates how to make this synthetic data statistically-relevant, as well as useful for algorithmic analysis and training (7,8). Such data show potential to improve data accessibility, model performance and eventually boost scientific progress. However, the creation and use of synthetic text comes with challenges.

*Key objective* of this perspective piece is to demystify the technology behind synthetic text generation and use by explaining its major challenges, both technical and ethical, and especially in relation to synthetic mental health text, as well as sketch the most promising methods to overcome them.

## 2. What is synthetic data?

In this work, we define synthetic text as artificially generated text based on use-case relevant context and that reflects the relevant meaning for statistical analysis in the intended context (including the training of, and analysis by AI).

There are multiple different ways other ways artificial text can be created. A straightforward way is to manipulate and modify the original text. For example, we can create a “noisy” version of the original text by replacing or swapping some words in it [for example, replacing some words with synonyms or words with close meaning from a pre-defined vocabulary or randomly (9,10)].

Another way is to automatically detect personal identifiable information (PII) in source text (such as names and addresses) and either completely remove them (anonymisation), replace them with place-holders [pseudonymisation, e.g., (11)] or sanitise them (obfuscate by means of semantic inference, i.e. replace with synonyms or homonyms) it [e.g., (12)].

Both of these approaches in themselves will not be sufficient to safeguard the privacy of mental health texts owing to the likelihood of sufficient identifying information (such as scenario, participant and location descriptions) surviving these processes.

One approach is to take a step back. Rather than modify source text to generate synthetic text, an AI model analyses the source text or/and any other additional information [meta data, images, video, sensor data, etc. (13,14)] to identify its meaning and other important contextual information to exclude the private information, and from this it generates new text from scratch which preserves these.

For example, the source text may contain the following sentence, “I walked-up the ticket office at Victoria Station but could not remember where I wanted to buy a ticket for”. The AI model would analyse this for meaning, i.e. that someone wanted to purchase a ticket for travel and that they expressed the sentiment of confusion. It would then generate a new sentence, such as “I went up to the ticket window but could not remember why I was there”. Such a rewriting preserves the validity of text for the automatic analysis (e.g., detection of confusion), but makes the description irrelevant to a situation of a particular individual neutralising the sensitive information.

In the following sections we will examine: first, how new text can be generated by means of language models; second, how this generation can be guided to ensure the preservation of relevant content and how the privacy of the original content can be preserved; and finally, discuss the ethical challenges of synthetic text usage such as benchmarking standard and bias.

## 3. Primer on language models

In its essence, AI language models perform language generation through forming sentences by picking words, one after another, from a learnt vocabulary. Their selection of words is driven by the probability distributions of words obtained by the analysis of large quantities of texts, aka training data. For example, we can compare how many times the words “book” and “bag” follow the phrase “He dropped the…” to get a relative probability distribution for them (e.g., 45% chance of “bag”, 10% chance of “book”) (15).

In addition, context must also be accounted for. In the transcript of an online chat “He dropped the…” may be more likely to be followed by the word “plans”, whereas in a legal text it may be “objection”.

In order to encode this combination of word probability distributions and textual contexts we make use of a concept called “word embedding” [e.g., BERT (16)]. Explained in simple terms, words are represented by sequences of numbers, these are in turn cross-referenced multiple times with weighted probabilities for their appearance in any given context as learnt from the training data.

This language modelling approach is sufficiently flexible to steer and adapt the generation process to particular situations and contexts. Using the examples above, it enables it to pick “bag”, “book”, “plans” or “objection” to follow “He dropped the…” as appropriate for the context.

Modern neural language models learn billions and trillions of parameters to predict word probabilities from large portions of unmoderated data coming from the Internet [e.g., GPT-3 (17)]. These models are publicly available online as generic generators via such libraries as Huggingface.

## 4. Algorithmic challenges

In order to be able to generate text which preserves the salient properties of the original, the language model needs to have access to relevant context information at the input stage. Currently, language models operate efficiently given well-defined but narrow contexts in such tasks as medical report generation when given images, tables or any other longer text (e.g., text to be summarised), etc. (18–20). These models are capable of distinguishing important information from the inputs which need to be preserved in the outputs (e.g., important figures from tables or medical image fragments relevant for diagnostic conclusions).

In the case of generating mental health text, the context includes many factors such as general diagnostic characteristics, personal physical and physiological idiosyncrasies, their prior experience and the immediate context of the conversation (e.g. where the conversation is taking place, mood and emotions of the interlocutor etc.). This indicates the crucial role of the human to distill and narrow the relevant information down to *control the text generation process* and hence produce valid mental health text for different scenarios (13). For example, the generation of clinical notes in the mental health domain has been attempted under the guidance of demographic information and keywords extracted from real text (21). Synthetic text generated this way has been shown useful as training data for an AI predicting mental health diagnoses.

Recent techniques to control text generation for large pre-trained publicly available language models have shown promising potential enabling the creation of computationally-tractable controllable models for multiple contexts (22,23).

As already mentioned, AI models, including language models, tend to memorise the training data. To illustrate this using an extreme case, should the training data contain one single example then the model would treat the probability of seeing this data in the task as equal to one. Following on from this, should a model give a phrase or sentence a probability of one, then it can be inferred that was in its training data. This is a simplistic explanation of model overfitting which could be used to gain knowledge of the training data. Hence the next challenge of synthetic text generation is how to prevent the language model from exhibiting such memorisation and *preserve privacy* of the respective individual.

A range of standard privacy protection techniques has now been applied to text generation. One of them, Differential Privacy (DP) (24) adds noise to the updates of model weights in such a way that the even singular training examples can not be inferred from outputs (25). An alternative way to train models without data sharing is proposed by the Federated Learning (FL) (26) technique. This technique allows us to learn global model weights without giving access for this global model to the local data. Models are learnt locally and are regularly merged into the global model at the next level of aggregation (27).

Note that these privacy preservation techniques do not represent a solution for the data sparsity issue and can not be seen as the replacement for the synthetic text generation techniques.

## 5. Ethical challenges

There are also a number of practical implications from the wider usage of synthetic text in AI training. The first one is the challenge of developing a *benchmarking standard* for synthetic text (28,29). As yet, in the clinical and mental health domains the utility of synthetic text has been evaluated for only some types of AI, such as automated diagnoses prediction (21) or named entity recognition (7). There are no best established practices or systematic criteria on how to assess synthetic data. Recently, AI evaluation techniques have turned to functional evaluation where model performance is benchmarked in a series of use case scenarios (30). An example for mental health synthetic text is measuring the changes in its clinical validity while modifying the input information, so that text generated for the input “bipolar” should contain “self-important” more often than the text generated for the input “depression”. The performance of AI trained on synthetic text will be systematically compared to that of AI trained on real text. Since model transparency is now a requirement for responsible AI development, these performance comparisons will be done at the level of model decision explanations (31). Overall, text generators need to be subject to systematic human monitoring at the model development stages.

The second challenge is the trade-off inherent in achieving the validity and utility necessary for the task against preserving input privacy (32). Taking a similar approach to that used for numerical data in statistics (33), methods are needed to assess these trade-offs on a per use case basis for the mental health domain. For example if we do not strike an appropriate balance, the danger in the case of rare mental health conditions (i.e., those affected by a paucity of training data due to a small sample size) is that the risk of patient re-identification may outweigh the benefits of reduced treatment costs from early automatic condition detection.

Another important ethical implication regarding synthetic data is *bias*. This becomes increasingly important in those mental health scenarios with naturally small sample sizes. We have seen that language models capture general trends in the training data and ignore the outliers. So that any bias in the existing data (an under-represented or mis-represented group) will be amplified in the synthetic text. Methods of controlled generation have been shown to be very efficient in addressing such bias issues since instances of detected bias can be corrected for by generating, under the control of human experts, compensatory examples with appropriate properties (34). Thus, they enhance and enrich the natural data.

This leads onto the possibility of creating banks or repositories of synthetic data of sufficient quality suitable for AI training in the mental health domain. In terms of cybersecurity there would be a risk of such datasets being interfered with or “poisoned” by malicious actors. This then introduces a need to “quality mark” such data to signify it has been produced to the required standard and is unchanged, a process which would involve a verification mechanism potentially using hashes or similar cryptographic mechanisms to verify data authenticity and integrity.

## 6. Future perspective

To conclude, in this perspective piece we want to emphasise the potential of synthetic text for advancing mental health research and practice through providing sufficient accessible data for AI training in this realm, and tried to demystify the process of natural language generation. In addition, we have shown that natural language generators are efficient tools which need to be guided and controlled by humans to produce adequate, ethically acceptable and statistically-relevant outputs. Current AI techniques provide efficient tools for controlling the generation process, as well as the fairness and privacy-preservation of the resulting text. More investigation is needed on the scope parameters which will guide the production of statistically-relevant outputs in particular use cases. There is a crucial lack of comprehensive frameworks to systematically assess the validity of synthetic text for further statistical analysis by AI. In the mental health domain where the seemingly opposing undercurrents of preserving patient privacy and achieving utility and validity must be carefully navigated, such assessment frameworks are particularly difficult to design. Needless to say whilst AI can offer good decision support it can not replace human expertise, especially in such a sensitive domain as mental health.

Beyond synthetic text being used for AI training, its potential can be investigated for synthetic statistical control populations in research settings (offering the prospect of providing reliable and inexpensive alternatives to recruiting human participants). Additionally, it could be used for data imputation in research to help address standard problems with missing and asymmetric data sampling.

Further integration of synthetic text needs to be supported by a mature legal framework as well as best practices of responsible AI development in the research community, with the latter accepted by and implemented by industry. The benefits of synthetic data may well prove to be a double-edged sword for industry, improving the models of those already in the market whilst due to reducing the reliance on real data opening other niches where competitors are able to flourish.

## Data Availability

The original contributions presented in the study are included in the article/supplementary material, further inquiries can be directed to the corresponding author/s.

## References

[1] Sawhney R Joshi H Gandhi S Jin D Shah RR. Robust suicide risk assessment on social media via deep adversarial learning. J Am Med Inform Assoc. (2021) 28:1497–506. 10.1093/jamia/ocab031. 33779728 PMC8279792

[2] Tsakalidis A Atzil-Slonim D Polakovski A Shapira N Tuval-Mashiach R Liakata M. Automatic identification of ruptures in transcribed psychotherapy sessions. Association for Computational Linguistics. In: Proceedings of the Seventh Workshop on Computational Linguistics and Clinical Psychology: Improving Access, Online. (2021), p. 122–8. 10.18653/v1/2021.clpsych-1.15

[3] Tsakalidis A Chim J Bilal IM Zirikly A Atzil-Slonim D Nanni F, et al. Overview of the CLPsych 2022 shared task: capturing moments of change in longitudinal user posts. Association for Computational Linguistics. In: Proceedings of the Eighth Workshop on Computational Linguistics and Clinical Psychology. Online. (2022). p. 184–98.

[4] Das S. Mental health helpline funded by royals shared users’ conversations. Observer Mental Health (2022). https://www.theguardian.com/society/2022/feb/19/mental-health-helpline-funded-by-royals-shared-users-conversations. Access date: 2 Aug 2022

[5] Abadi M Chu A Goodfellow I McMahan HB Mironov I Talwar K, et al. Deep learning with differential privacy. Association for Computing Machinery. In: Proceedings of the 2016 ACM SIGSAC Conference on Computer and Communications Security, Vienna, Austria. (2016). p. 308–18. 10.1145/2976749.2978318

[6] Walsh T. Will AI end privacy? How do we avoid an Orwellian future. AI Soc (2022) 1:3. 10.1007/s00146-022-01433-y

[7] Li J Zhou Y Jiang X Natarajan K Pakhomov SV Liu H, et al. Are synthetic clinical notes useful for real natural language processing tasks: a case study on clinical entity recognition. J Am Med Inform Assoc (2021) 28:2193–2201. 10.1093/JAMIA/OCAB112. 34272955 PMC8449609

[8] Nikolenko SI, Springer optimization and its applications. Synthetic data for deep learning. Springer Cham 174 (2021). 10.1007/978-3-030-75178-4

[9] Wei J Zou K. EDA: easy data augmentation techniques for boosting performance on text classification tasks. Association for Computational Linguistics In: Findings of the Association for Computational Linguistics: ACL-IJCNLP 2022, Online (2022). pp 968–88, 10.18653/V1/2021.FINDINGS-ACL.84

[10] Feng SY Gangal V Wei J Chandar S Vosoughi S Mitamura T, et al. A survey of data augmentation approaches for NLP. In: *Findings of the Association for Computational Linguistics: ACL-IJCNLP 2022*, Online (2022). p. 968–88. 10.18653/V1/2021.FINDINGS-ACL.84

[11] Stubbs A Uzuner Ö. Annotating longitudinal clinical narratives for de-identification: the 2014 i2b2/UTHealth corpus. J Biomed Inform (2015) 58:S20–9. 10.1016/J.JBI.2015.07.020 26319540 PMC4978170

[12] Rodriguez-Garcia M Batet M Sánchez D. A semantic framework for noise addition with nominal data. Knowledge-Based Syst (2017) 122:103–18. 10.1016/J.KNOSYS.2017.01.032

[13] Keskar NS Mccann B Varshney LR Xiong C Socher R Research S. CTRL: a conditional transformer language model for controllable generation. CoRR. abs/1909.05858 (2019).

[14] Li G Zhu L Liu P Reler YY. Entangled transformer for image captioning In: *Proceedings of 2019 IEEE/CVF International Conference on Computer Vision (ICCV)*. IEEE: Seoul, Korea (2019). pp 8927–8936. 10.1109/ICCV.2019.00902

[15] Jurafsky D Martin JH, Speech, language processing: an introduction to natural language processing, computational linguistics„ speech recognition. 3rd ed (2021). https://web.stanford.edu/~jurafsky/slp3/ed3book_jan122022.pdf. Access date: 2 Aug 2022

[16] Devlin J Chang M-W Lee K Toutanova K. {BERT}: pre-training of deep bidirectional transformers for language understanding. In: *Proceedings of the 2019 Conference of the North American Chapter of the Association for Computational Linguistics: Human Language Technologies, Volume 1*. Minneapolis, USA: Long and Short Papers. (2019). p. 4171–86. 10.18653/v1/N19-1423

[17] Brown TB Mann B Ryder N Subbiah M Kaplan J Dhariwal P, et al. Language models are few-shot learners. *Adv Neural Inf Process Syst*. (2020) 33:1877–901.

[18] Chen J Guo H Yi K Li B Elhoseiny M. VisualGPT: data-efficient adaptation of pretrained language models for image captioning In: *Proceedings 2022 IEEE/CVF Conference on Computer Vision and Pattern Recognition (CVPR)*. New Orleans, LA, USA: IEEE (2022). pp 18009-18019. doi: 10.1109/CVPR52688.2022.01750

[19] Wang M Wang M Yu F Yang Y Walker J Mostafa J. A systematic review of automatic text summarization for biomedical literature, EHRs. J Am Med Inform Assoc (2021) 28:2287–97. 10.1093/jamia/ocab143 34338801 PMC8449627

[20] Wu H-Y Zhang J Ive J Li T Gupta V Chen B, et al. Medical scientific table-to-text generation with human-in-the-loop under the data sparsity constraint [preprint] (2022). Available at doi:10.48550/ARXIV.2205.12368

[21] Ive J Viani N Kam J Yin L Verma S Puntis S, et al. Generation, evaluation of artificial mental health records for natural language processing. NPJ Digit Med (2020) 3:69. 10.1038/s41746-020-0267-x 32435697 PMC7224173

[22] Dathathri S Madotto A Lan J Hung J Frank E Molino P, et al. Plug, play language models: a simple approach to controlled text generation (2020). In: Proceedings International Conference on Learning Representations. Online (2020).

[23] Lester B Al-Rfou R Constant N. The power of scale for parameter-efficient prompt tuning. Association for Computational Linguistics (2021). p. 3045–59.

[24] Dwork C. Differential privacy. Automata, Languages and Programming. ICALP. In: Bugliesi, M., Preneel, B., Sassone, V., Wegener, I. editors. Lecture Notes in Computer Science. Springer, Berlin, Heidelberg, vol 4052 (2006) 10.1007/11787006_1

[25] Ponomareva N Bastings J Vassilvitskii S. Training text-to-text transformers with privacy guarantees. In: *Findings of the Association for Computational Linguistics: ACL* 2022, Online (2022). p. 2182–93. 10.18653/v1/2022.findings-acl.171

[26] McMahan HB Moore E Ramage D Hampson S AgüeraAg B Arcas A, Communication-efficient learning of deep networks from decentralized data. In: *Proceedings of the 20th International Conference on Artificial Intelligence and Statistics (AISTATS)*. Fort Lauderdale, Florida, USA: ML Research Press. (2017). pp 1273–1282.

[27] Lin BY He C Ze Z Wang H Hua Y Dupuy C, et al. FedNLP: benchmarking federated learning methods for natural language processing tasks. In *Findings of the Association for Computational Linguistics: NAACL 2022*. Seattle, USA: Association for Computational Linguistics (2022). p. 157–75.

[28] Chen RJ Lu MY Chen TY Williamson DF Mahmood F. Synthetic data in machine learning for medicine and healthcare. Nat Biomed Engng (2021) 5:493–7. 10.1038/s41551-021-00751-8 34131324 PMC9353344

[29] Zhang A Xing L Zou J Wu JC. Shifting machine learning for healthcare from development to deployment and from models to data. Nat Biomed Engng (2022) 2022:1–16. 10.1038/s41551-022-00898-y PMC12063568 35788685

[30] Ribeiro MT Wu T Guestrin C Singh S. Beyond accuracy: behavoral testing of NLP models with checklist. Association for Computational Linguistics In: *Proceedings of the 58th Annual Meeting of the Association for Computational Linguistics*. (2020). p. 4902–12. 10.18653/v1/2020.acl-main.442

[31] Wiegreffe S Marasovic A. Teach me to explain: a review of datasets for explainable NLP In: *Proceedings of the Neural Information Processing Systems Track on Datasets and Benchmarks, Online*. (2021). Vol. abs/2102.12060

[32] Lison P Pilán I Sanchez D Batet M Øvrelid L. Anonymisation models for text data: state of the art, challenges and future directions. Association for Computational Linguistics (2021). p. 4188–203. 10.18653/v1/2021.acl-long.323

[33] Abowd JM Schmutte IM. An economic analysis of privacy protection and statistical accuracy as social choices. Am Econ Rev (2019) 109:171–202. 10.1257/AER.20170627

[34] Meade N Poole-Dayan E Reddy S. An empirical survey of the effectiveness of debiasing techniques for pre-trained language models. In: *Proceedings of the 60th Annual Meeting of the Association for Computational Linguistics Volume 1*. Dublin, Ireland: Long Papers. (2022). p. 1878–98. 10.18653/v1/2022.acl-long.132

